# Potential Use of VYN202, a Novel Small Molecular Bromodomain and Extra-Terminal Inhibitor, in Mitigating Secondhand Smoke (SHS)-Induced Pulmonary Inflammation

**DOI:** 10.3390/cimb47121062

**Published:** 2025-12-18

**Authors:** Katelyn A. Sturgis, Benjamin D. Davidson, Andrew W. Richardson, Olivia Hiatt, Blake C. Edwards, Ethan P. Evans, Carrleigh Campbell, Jack H. Radford, Juan A. Arroyo, Benjamin T. Bikman, Paul R. Reynolds

**Affiliations:** Department of Cell Biology and Physiology, Brigham Young University, Provo, UT 84602, USA

**Keywords:** inflammation, lung, tobacco, secondhand smoke

## Abstract

Inflammation underpins pulmonary disease progression during tobacco smoke exposure, which may culminate in irreversible pulmonary disease. While primary smoke poses a notable risk, nearly half of the US population is also susceptible due to frequent exposure to secondhand smoke (SHS). In the present study, we assessed the potential role of VYN202, a novel small molecular bromodomain and extra-terminal inhibitor, as a possible means of attenuating SHS-mediated inflammation. We exposed wild-type mice to an acute time course of room air (RA), SHS via a nose-only delivery system (Scireq Scientific, Montreal, Canada), or to both SHS and 10 mg/kg VYN202 (efficacious dose from prior inflammatory models) via oral gavage three times a week. Specific smoke exposure delivery to mice involved SHS from two cigarettes over 10 min, equilibration in room air for 10 min, followed by exposure to SHS from one cigarette for an additional 10 min, for a total SHS exposure of 20 min per day, five days a week for 30 days. We evaluated leukocyte abundance and the secretion of inflammatory mediators in bronchoalveolar lavage fluid (BALF). We also assessed general morphology via histology staining and the activation of receptor tyrosine kinase (RTK) family members. While standard hematoxylin and eosin (H&E) staining resulted in unchanged morphology, SHS-mediated increases in BALF protein abundance, total cellularity, and percent PMNs were attenuated with concomitant administration of VYN202. We also discovered SHS-induced activation of RTKs that were pro-inflammatory (JAK1, JAK3, ABL1, and ACK1), as well as RTKs related to endothelial and vascular remodeling (VEGFR3, VEGFR2, EphB4, EphB6, and FAK). Furthermore, inflammatory cytokines including GCSF, IFN-γ, IL-12p70, IL-17A, LIX, and TNF-α were all augmented by SHS exposure. Despite SHS exposure, each of these RTKs and cytokines/chemokines was significantly attenuated by VYN202. In summary, inflammatory responses induced by SHS exposure were mitigated by VYN202. These data reveal fascinating potential for the utility of VYN202 in lessening smoke-induced pulmonary exacerbations.

## 1. Introduction

Secondhand smoke (SHS) exposure is a well-documented environmental hazard linked to acute and chronic respiratory diseases, including chronic obstructive pulmonary disease (COPD) [1]. SHS consists of thousands of toxic compounds, including reactive oxidants, polycyclic aromatic hydrocarbons, and particulate matter, which elicit immune responses upon inhalation [2]. While acute exposure triggers a transient inflammatory response primarily via polymorphonuclear leukocyte (PMN) recruitment and activation [3], repeated and prolonged exposure promotes persistent immune activation, tissue remodeling, and epigenetic alterations contributing to chronic lung pathology [4]. The use of rodent SHS models has demonstrable value in modeling short term consequences of exposure as well as COPD pathology [5]. Among the molecular pathways disrupted by SHS, receptor tyrosine kinase (RTK) signaling and inflammatory cytokine secretion play pivotal roles in disease progression [4]. In recent years, epigenetic regulators such as the bromodomain and extra-terminal (BET) protein family have emerged as potential therapeutic targets due to their role in transcriptional control of inflammatory gene expression. This study explores the mechanistic link between SHS exposure, RTK dysfunction, and epigenetic reprogramming, focusing on BET inhibition as a promising strategy for mitigating inflammatory lung diseases [6].

Acute SHS exposure rapidly activates the innate immune system, leading to the recruitment of neutrophils and macrophages into the lung parenchyma [7]. This inflammatory response is primarily driven by the release of pro-inflammatory cytokines such as tumor necrosis factor-alpha (TNF-α), interleukin-1 beta (IL-1β), and granulocyte colony-stimulating factor (G-CSF) [8]. In healthy individuals, acute inflammation resolves through anti-inflammatory mechanisms, including the secretion of IL-10 and the activation of tissue-resolving macrophages [9]. However, chronic exposure to SHS disrupts this balance, leading to sustained activation of inflammatory pathways and progressive lung tissue damage [10].

The transition from acute inflammation to chronic lung disease is marked by dysregulated RTK signaling. RTKs such as ABL1, JAK1/3, and VEGFR2 are upregulated in response to SHS, perpetuating the inflammatory response and promoting fibrotic remodeling [11]. Long-term SHS exposure drives alveolar destruction, peribronchial fibrosis, and airway narrowing, hallmark features of COPD [12]. Furthermore, chronic inflammation enhances susceptibility to respiratory infections and exacerbates oxidative damage, compounding disease severity. Understanding the molecular events driving SHS-induced inflammation and COPD progression is essential for developing targeted therapeutic strategies [13].

Epigenetic modifications, including DNA methylation, histone modifications, and chromatin remodeling, regulate RTK activity and inflammatory gene expression [14]. SHS exposure induces widespread epigenetic alterations that disrupt normal lung homeostasis. For instance, DNA hypermethylation of anti-inflammatory genes leads to sustained immune activation, while histone acetylation at pro-inflammatory loci enhances cytokine secretion [15]. These epigenetic changes contribute to the persistent activation of NF-κB and STAT3 pathways, amplifying the inflammatory response and promoting disease progression [16]. RTK-mediated signaling is particularly susceptible to epigenetic modulation. In SHS-exposed lung tissues, aberrant histone acetylation enhances the transcription of RTKs such as ABL1 and JAK1, perpetuating inflammatory signaling [17]. Likewise, chromatin remodeling at cytokine gene loci facilitates sustained IL-6, IL-17A, and TNF-α secretion, further reinforcing the chronic inflammatory state. Accordingly, targeting key epigenetic regulators represents a promising therapeutic approach due to epigenetics’ central role in SHS and lung disease progression [18].

The BET protein family, which includes BRD2, BRD3, BRD4, and BRDT, serves as a key epigenetic transcriptional regulator by recognizing acetylated histones and recruiting transcriptional machinery to active gene promoters [19]. BET proteins are critical mediators of inflammatory gene expression, particularly through their interaction with NF-κB and STAT signaling pathways [20]. BET proteins facilitate the sustained transcription of pro-inflammatory cytokines and RTKs in SHS-induced lung inflammation, exacerbating disease pathology [21]. BET inhibitors (BETis), such as JQ1 and I-BET762, have demonstrated significant potential in suppressing inflammatory gene expression in preclinical models of respiratory diseases [22]. These inhibitors function by competitively displacing BET proteins from chromatin, leading to transcriptional repression of inflammatory mediators [23]. In experimental COPD models, BET inhibition has been shown to reduce IL-6, TNF-α, and IL-17A levels, alleviating airway inflammation and fibrosis [24]. While pan-BET inhibitors like JQ1 have demonstrated broad suppression of inflammatory genes (e.g., IL-6, TNF-α) in smoke-exposed models [25], selective BD2 inhibitors such as VYN202 may offer improved safety profiles by targeting specific epigenetic interactions [26]. Furthermore, BETis have been reported to attenuate RTK-mediated signaling cascades, providing a dual mechanism for controlling immune activation and epithelial dysfunction [27].

This study evaluated the effects of an orally administered small-molecule BET inhibitor, VYN202, in an acute SHS-induced mouse model. VYN202, a small molecule antagonist of the BET family, binds to BD2 motifs of BET proteins (EC50_BD2 BRD4_: 1 nM; EC50_BD1 BRD4_: >10,000 nM) and has demonstrated activity in downregulating key inflammatory pathways relevant to autoimmune diseases in ex vivo human cell/tissue cultures and in vivo rodent models. In addition, VYN202 has been demonstrated to be an effective inhibitor via BRD4 displacement assays and transcriptomic changes in BET target genes (I. Stuart, personal communication, February 2025). VYN202 is currently being clinically evaluated as an oral treatment for moderate-to-severe plaque psoriasis.

In summary, SHS exposure induces a complex interplay of inflammatory responses, epigenetic modifications, and RTK dysregulation, culminating in progressive lung damage. Epigenetic alterations are key drivers of persistent inflammation, with BET proteins playing a pivotal role in transcriptional activation of inflammatory genes. BET inhibitors offer a promising therapeutic avenue by targeting the epigenetic machinery that sustains inflammation, thereby providing a novel approach for mitigating SHS-induced lung pathology.

## 2. Materials and Methods

### 2.1. Animals

Twelve-week-old female WT mice (obtained from Jackson Laboratories; Bar Harbor, ME, USA) were generated on a C57BL/6 background. While an analysis of both sexes is preferred due to the plausibility of sex-dependent differences, we selected females only in the current project due to pronounced inflammatory outcomes demonstrated previously [28,29,30]. Rodents were supplied with food and water ad libitum in a specific pathogen-free facility and maintained on a 12 h light–dark cycle. Select mice (n = 8) were exposed to SHS generated from 3R4F research cigarettes from Kentucky Tobacco Research and Development Center, University of Kentucky, using a nose-only exposure system (InExpose System, Scireq, Montreal, QC, Canada) as outlined previously [31]. SHS was generated by passive sidestream burning of 3R4F cigarettes and collected via the Scireq InExpose delivery system, which provided nose-only exposure to diluted SHS. Cigarette smoke was not delivered directly as mainstream smoke. Instead, smoke was allowed to equilibrate in ambient air before being directed into the exposure tower. Based on prior system calibration and comparable protocols, the estimated total suspended particulate matter (TSP) during exposure was approximately 100–150 mg/m^3^, consistent with our previously published SHS rodent models. Mice were exposed to SHS from two cigarettes over 10 min, allowed to equilibrate in room air for 10 min, then exposed to smoke from one cigarette for an additional 10 min, for a total SHS exposure of 20 min per day. This procedure was repeated five days a week for 30 days and compared to control mice (n = 8) that were similarly restrained and exposed to room air (RA) only. A detailed description of the SHS study design and its use in modeling pulmonary compromise has been described extensively by our group [31,32].

According to previously published reports, the SHS challenge was tolerated well in terms of toxicity and was delivered at an acceptable level of particulate density concentration [33]. A third group of mice (n = 8) was exposed to SHS and administered 10 mg/kg VYN202 suspended in PBS + 5% DMSO via oral gavage thrice weekly. A final group of mice (n = 8) was exposed to SHS and 5% DMSO only. In accordance with an initial pilot dose curve study (not provided), the VYN202 administration program was designed following toxicity and pharmacokinetic screens that revealed no adverse effects, weight deviations (body and organ), and detectable anti-inflammatory characteristics. After the exposure, and in line with previously published protocols, mice were sacrificed. The right lung was tied off with suture string and the left lung was lavaged to collect bronchoalveolar lavage fluid (BALF). Unlavaged right lobes were fixed with 4% paraformaldehyde and processed for histology samples or resected and immediately snap-frozen in liquid nitrogen for analysis of markers in total lung lysates [34,35]. Sections of lung tissue (5 mm each) were immunostained to qualitatively assess relative macrophage quantity. Lung samples were specifically stained for Mac-3 (Cat# 550292; 1:50; BD Biosciences, San Jose, CA, USA), an antibody that recognizes the 110 kDa Mac-3 protein expressed by mononuclear phagocytes, and a goat anti-rat secondary antibody (Cat# sc-2032; Santa Cruz Biotechnology, Santa Cruz, CA, USA). All animal studies were conducted in two independent experimental replicates, each performed under identical conditions. The findings presented here include data from both replicates unless otherwise specified. All experimental animal studies were approved by the Institutional Animal Care and Use Committee (IACUC) at Brigham Young University, and all methods were carried out in accordance with relevant animal guidelines and regulations. The reporting of animal methods was in accordance with ARRIVE guidelines for reporting animal experiments.

### 2.2. Bronchoalveolar Lavage Fluid (BALF) Analyses for Cellularity or Cytkines/Chemokines

On the sacrifice date, BALF was procured and evaluated as outlined previously [36]. Left lobes were isolated and a 20-gauge cannula was inserted into the trachea. PBS was used to lavage the lobes four times and then extracted. The resulting BALF was collected and centrifuged at 4 °C. Following centrifugation, the pellet was resuspended in 500 μL of sterile PBS; total cell counts were measured manually using a hemocytometer where counts were performed at least three times and averaged. A portion of the resuspension was fixed on a histological slide via cytospin for differential staining. The percentage of polymorphonuclear (PMN) cells was determined by counting PMNs among 200 total cells in each slide differentially stained. Counts were repeated at least three times and averaged. BALF was also screened for cytokines and chemokines using a mouse Inflammation Array C3 (RayBiotech, Norcross, GA, USA), as demonstrated by our lab previously [32]. Briefly, 15 μg of total BALF protein per sample (RA control, SHS, and SHS + VYN202 for a total of n = 6 per group) was collected and divided to create two sample pools, each with a concentration of 125 μg/mL (BALF samples from 3 animals were pooled for each blot; n = 3 animals per blot). Two sample pools per group were added to individual membranes and incubated overnight. Biotinylated antibodies were added to each membrane, followed by a final incubation with a streptavidin-conjugated fluorescent label (Thermo Fisher Scientific, Waltham, MA, USA) to detect molecule expression. Membranes were imaged using the Odyssey DLx Near-Infrared Fluorescence Imaging System (LI-COR, Lincoln, NE, USA). Results were generated as ratios between the marker of interest and the kit’s positive controls via quantitative analysis using Image J (version 1.54, U.S. National Institutes of Health, Bethesda, MD, USA).

### 2.3. Receptor Tyrosine Kinase (RTK) Superfamily Analyses

Lung lysates were screened for RTK-related molecules (Figures 2–6) with a mouse RTK Phosphorylation Array C1 (RayBiotech, Norcross, GA, USA). In this case, 125 μg of total protein lysate per sample (RA control, SHS, and SHS + VYN202 for a total of n = 6 per group) was collected and divided to create two sample pools, each with a concentration of 500 μg/mL (protein samples from 3 animals were pooled for each blot; n = 3 animals per blot). Details regarding incubating membranes, biotinylated antibodies, and streptavidin-conjugated fluorescent labeling to detect molecule expression were identical to the procedures outlined for BALF assessments described above. Membranes were imaged using the same Odyssey DLx Near-Infrared Fluorescence Imaging System and ratios between the marker of interest and the positive control were presented following analysis using Image J as noted.

### 2.4. Pulmonary Function Testing

We used a separate cohort of mice to assess lung function. Pulmonary mechanics were assessed using the FlexiVent FX system (SCIREQ) equipped with the NPFE module. Mice were anesthetized with intraperitoneal ketamine/xylazine (100/10 mg/kg), tracheostomized, and connected to the ventilator via a 22 G cannula. Pancuronium bromide (0.8 mg/kg) was administered to prevent spontaneous breathing. Ventilation settings included a tidal volume of 10 mL/kg, 150 breaths/min, and 3 cmH_2_O PEEP. Standardized maneuvers—including Deep Inflation, Snapshot-150, Quick Prime, and NPFE—were performed in triplicate per manufacturer protocol as already described [31]. Mice were euthanized following completion of the procedure.

### 2.5. Statistical Analysis

Data were shown as means ± SE. Differences in BALF cytokines or lung protein markers were determined between control and treated samples. One-way ANOVA tests were used to compare protein expression between the groups, and significant differences between groups were noted at *p* < 0.05. Statistical analysis was performed with GraphPad Prism 8.0 software.

## 3. Results

**Figure 1:**
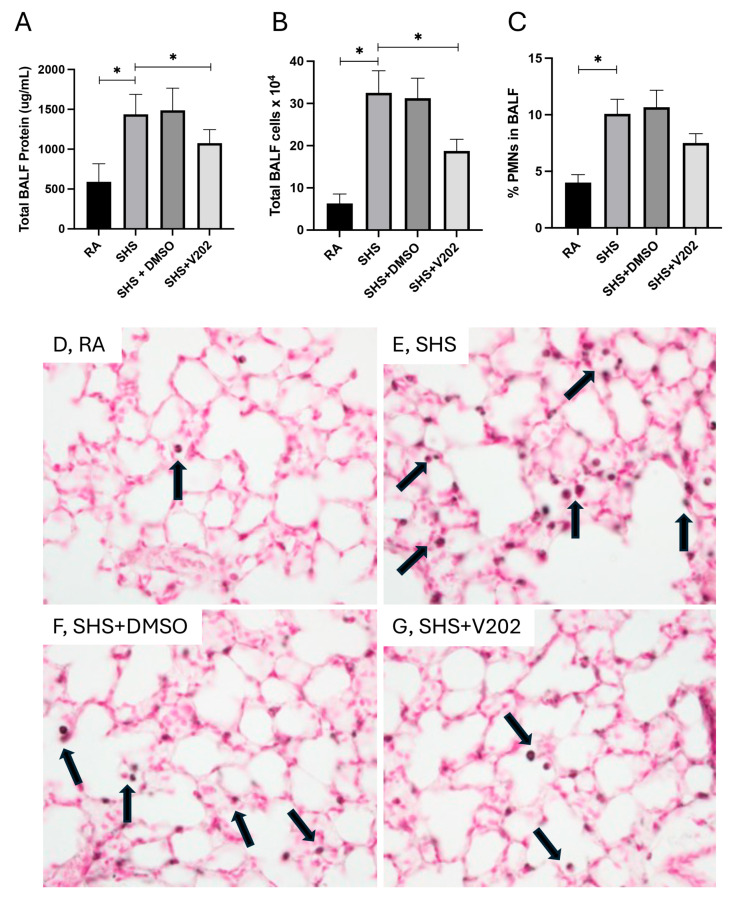
VYN202 attenuates SHS-induced alveolar barrier disruption and leukocyte infiltration. Total BALF protein concentration (**A**), total BALF leukocyte counts (**B**), and percentage of PMNs in BALF (**C**) were measured following 30 days of room air (RA), SHS, or SHS + VYN202 exposure. Exposure to RA or SHS occurred five days per week for 30 days and where indicated, VYN202 was administered by gavage thrice weekly for 30 days. SHS significantly increased all parameters relative to RA; VYN202 significantly reduced each relative to SHS alone. Significance is noted as *p* < 0.05. SHS + DMSO (vehicle only control) did not significantly alter SHS-induced metrics. Representative images of Mac3 immunohistochemistry, a marker for macrophages, in lung sections from RA (**D**), SHS (**E**), SHS + DMSO (**F**), and SHS + VYN202 (**G**) groups. Arrows indicate perivascular and peribronchiolar Mac3-positive macrophages. SHS exposure increased the abundance of Mac3-positive macrophages compared to RA, while VYN202 treatment reduced Mac3 abundance relative to SHS alone. Significance is noted as * *p* < 0.05.

To evaluate pulmonary inflammation following SHS exposure, we quantified total protein content, total leukocyte quantity, and the proportion of polymorphonuclear neutrophils (PMNs) in BALF. SHS exposure significantly elevated total BALF protein levels compared to room air (RA) controls (Figure 1A; *p* < 0.05), indicative of increased alveolar-capillary barrier permeability. Administration of VYN202 during SHS exposure markedly reduced BALF protein levels compared to SHS exposure alone (Figure 1A). Similarly, total leukocyte counts in the BALF were significantly higher in SHS-exposed mice relative to RA controls (Figure 1B; *p* < 0.05), and treatment with VYN202 significantly attenuated leukocyte accumulation (Figure 1B). Analysis of leukocyte composition revealed that the percentage of PMNs was also increased in the SHS group compared to RA controls (Figure 1C; *p* < 0.05), consistent with neutrophilic inflammation. VYN202 administration reduced PMN percentages compared to SHS alone, suggesting that VYN202 mitigates SHS-induced neutrophilic infiltration (Figure 1C). Importantly, vehicle-alone data involving 5% DMSO did not significantly alter these metrics. These findings demonstrate that VYN202 effectively reduces SHS-induced alveolar protein leakage, leukocyte recruitment, and neutrophil-driven pulmonary response.

**Figure 2:**
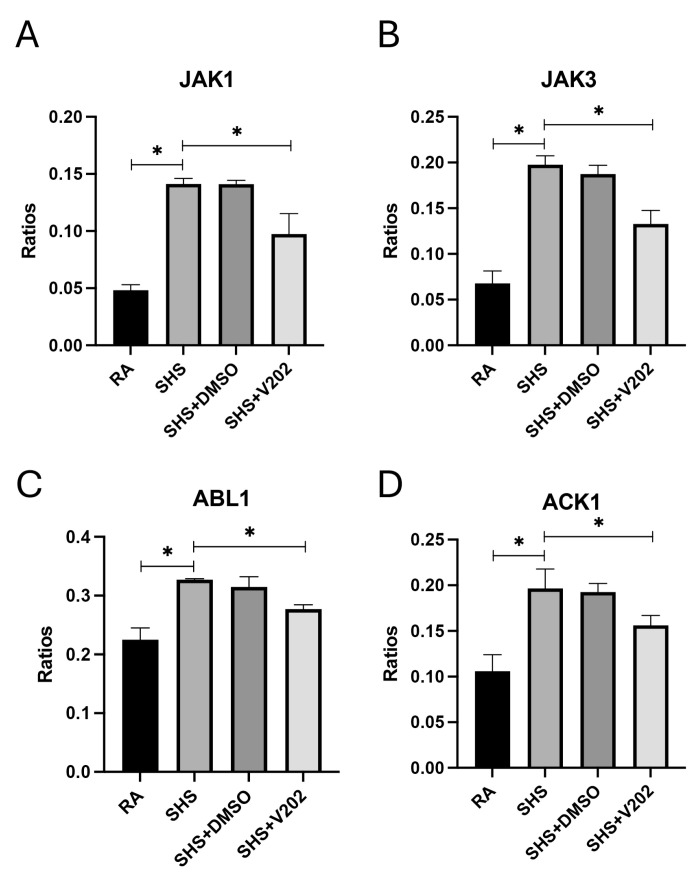
VYN202 suppresses SHS-induced activation of pro-inflammatory RTKs. Activation ratios of JAK1 (**A**), JAK3 (**B**), ABL1 (**C**), and ACK1 (**D**) were quantified following exposure conditions. SHS significantly elevated activation of all RTKs compared to RA, while VYN202 treatment significantly reduced activation relative to SHS. Exposure to RA or SHS occurred five days per week for 30 days and where indicated, VYN202 was administered by gavage thrice weekly for 30 days. Significance is noted as *p* < 0.05. SHS + DMSO (vehicle only control) did not significantly alter SHS-induced marker expression. Significance is noted as * *p* < 0.05.

As was demonstrated previously [37,38,39], there were no observable gross architectural differences observed by H&E staining when comparing lung samples from any of the groups (Figure 1D–G); however, VYN202 was sufficient to ameliorate SHS-induced leukocyte extravasation and alterations in lung function tests including forced expiratory volume in 0.1 s (FEV0.1) and forced expiratory volume at peak expiratory flow (FEV at PEF; Supplementary Figure S1). As there were no observable morphology changes, our general focus remained dissecting potential acute differences in the expression of molecular markers that regulate lung responses to SHS. To assess the effects of SHS exposure on pro-inflammatory signaling, we quantified activation of receptor tyrosine kinases (RTKs) implicated in immune hyperactivation (Supplementary Figure S1). SHS exposure significantly elevated the phosphorylation of JAK1, JAK3, ABL1, and ACK1 compared to RA controls (Figure 2A–D; *p* < 0.05 for each). These RTKs amplify inflammatory cascades by promoting cytokine production and leukocyte recruitment. Notably, VYN202 treatment during SHS exposure markedly reduced the activation of all four RTKs (Figure 2A–D). While VYN202 did not restore phosphorylation ratios to levels observed in RA controls, there were significant decreases in the activation of each candidate despite SHS exposure (Figure 2A–D). Importantly, vehicle-alone data involving 5% DMSO did not elicit pro- or anti-inflammatory effects. These findings indicate that VYN202 mitigates SHS-induced immune hyperactivation, likely by dampening RTK-driven signaling pathways that sustain chronic pulmonary inflammation.

**Figure 3:**
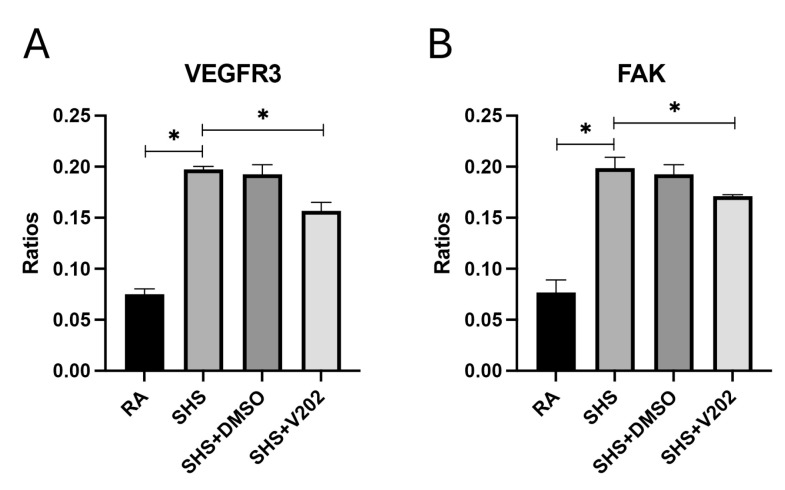
VYN202 mitigates SHS-induced activation of vascular remodeling RTKs. Phosphorylation levels of VEGFR3 (**A**) and FAK (**B**) were assessed in lung tissues. SHS exposure increased activation relative to RA; VYN202 administration significantly attenuated these increases. Exposure to RA or SHS occurred five days per week for 30 days and where indicated, VYN202 was administered by gavage thrice weekly for 30 days. Significance is noted as *p* < 0.05. SHS + DMSO (vehicle only control) did not significantly alter SHS-induced marker expression. Significance is noted as * *p* < 0.05.

We next examined RTKs involved in regulating vascular integrity and remodeling following SHS exposure. SHS significantly increased the activation of VEGFR3 and focal adhesion kinase (FAK) compared to RA controls (Figure 3A,B; *p* < 0.05 for each). These RTKs are key mediators of endothelial barrier dysfunction, capillary leakage, fibrosis, and inflammatory cell infiltration during pulmonary injury. VYN202 administration during SHS exposure significantly reduced VEGFR3 and FAK activation, notwithstanding SHS exposure (Figure 3A,B). These findings suggest that VYN202 may preserve vascular integrity and limit fibrotic and inflammatory processes by suppressing SHS-induced activation of vascular remodeling pathways.

**Figure 4:**
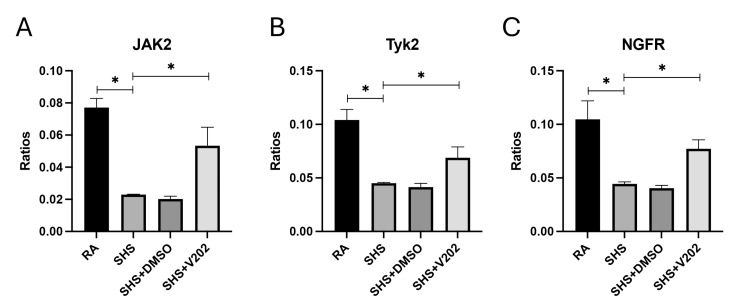
VYN202 partially restores SHS-suppressed anti-inflammatory and immune-regulatory RTK activation. Activation of JAK2 (**A**), Tyk2 (**B**), and NGFR (**C**) was reduced by SHS relative to RA. VYN202 treatment significantly elevated activation relative to SHS, though levels remained lower than RA controls. Exposure to RA or SHS occurred five days per week for 30 days and where indicated, VYN202 was administered by gavage thrice weekly for 30 days. Significance is noted as *p* < 0.05. SHS + DMSO (vehicle only control) did not significantly alter SHS-induced marker expression. Significance is noted as * *p* < 0.05.

Next, we assessed RTKs with known anti-inflammatory and immune-regulatory functions. SHS exposure significantly suppressed the activation of JAK2, Tyk2, and NGFR compared to RA controls (Figure 4A–C; *p* < 0.05 for each). These RTKs are critical for counterbalancing inflammatory responses, and their suppression is associated with amplified cytokine production and prolonged immune activation. VYN202 treatment during SHS exposure partially reversed the suppression of SHS-induced JAK2, Tyk2, and NGFR, although phosphorylation remained lower than RA controls. Importantly, activation levels were significantly elevated compared to SHS alone (Figure 4A–C). Importantly, vehicle-alone data involving 5% DMSO did not elicit pro- or anti-inflammatory effects. These findings suggest that, in addition to suppressing pro-inflammatory pathways, VYN202 may help reestablish regulatory signaling mechanisms that restrain excessive immune activation during SHS-induced pulmonary inflammation.

**Figure 5:**
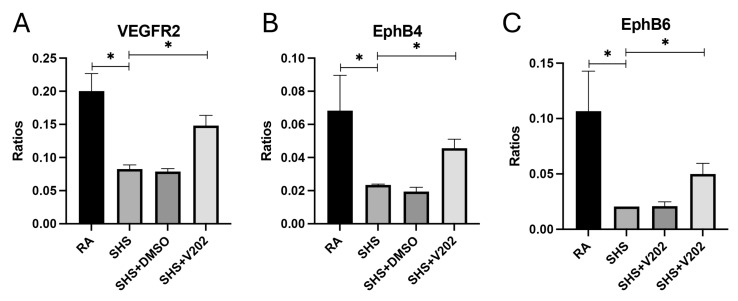
VYN202 modulates SHS-induced suppression of endothelial stability RTKs. Phosphorylation levels of VEGFR2 (**A**), EphB4 (**B**), and EphB6 (**C**) were significantly decreased by SHS exposure. VYN202 treatment partially restored activation relative to SHS alone. Exposure to RA or SHS occurred five days per week for 30 days and where indicated, VYN202 was administered by gavage thrice weekly for 30 days. Significance is noted as *p* < 0.05. SHS + DMSO (vehicle only control) did not significantly alter SHS-induced marker expression. Significance is noted as * *p* < 0.05.

To further evaluate vascular signaling disruptions following SHS exposure, we examined RTKs involved in maintaining endothelial integrity. SHS significantly suppressed the activation of VEGFR2, EphB4, and EphB6 compared to RA controls (Figure 5A–C; *p* < 0.05 for each). These RTKs promote vascular stability, and their inhibition is associated with exacerbated endothelial dysfunction, increased vascular permeability, and enhanced inflammatory cell infiltration. VYN202 treatment during SHS exposure partially restored SHS-mediated inactivation of VEGFR2, EphB4, and EphB6, with phosphorylation levels remaining lower than RA controls but significantly elevated compared to SHS alone (Figure 5A–C). These results suggest that VYN202 may help preserve vascular health by mitigating SHS-induced suppression of key endothelial and vascular stability regulators.

**Figure 6:**
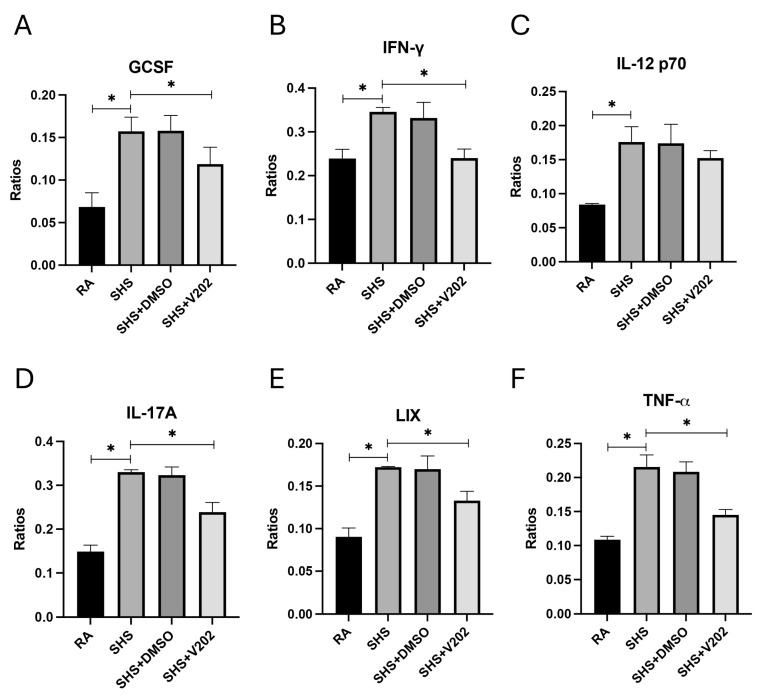
VYN202 attenuates SHS-driven pro-inflammatory cytokine and chemokine production. Lung levels of GCSF (**A**), IFN-γ (**B**), IL-12p70 (**C**), IL-17A (**D**), LIX (**E**), and TNF-α (**F**) were significantly elevated following SHS exposure. VYN202 significantly reduced expression of each mediator relative to SHS. Exposure to RA or SHS occurred five days per week for 30 days and where indicated, VYN202 was administered by gavage thrice weekly for 30 days. Significance is noted as *p* < 0.05. SHS + DMSO (vehicle only control) did not significantly alter SHS-induced marker expression. Significance is noted as * *p* < 0.05.

We then measured key pro-inflammatory cytokines and chemokines implicated in lung injury and immune dysregulation in BALF to assess downstream inflammatory consequences of SHS exposure. SHS exposure significantly elevated the expression of granulocyte colony-stimulating factor (GCSF), interferon-gamma (IFN-γ), interleukin-12p70 (IL-12p70), interleukin-17A (IL-17A), lipopolysaccharide-induced CXC chemokine (LIX), and tumor necrosis factor-alpha (TNF-α) compared to RA controls (Figure 6A–F; *p* < 0.05 for each). These mediators promote neutrophil recruitment and T cell activation, disrupt epithelial barriers, and cause fibrotic remodeling in smoke-related pulmonary diseases. Although levels remained elevated compared to RA controls, VYN202 treatment during SHS exposure significantly attenuated the abundance of each inflammatory mediator relative to SHS alone (Figure 6A–F). Importantly, vehicle-alone data involving 5% DMSO did not elicit pro- or anti-inflammatory effects. These findings suggest that VYN202 mitigates SHS-driven amplification of inflammatory signaling networks, potentially limiting sustained immune activation, tissue damage, and disease progression in smoke-exposed lungs.

## 4. Discussion

SHS exposure remains a significant public health concern, contributing to the development and progression of chronic pulmonary diseases such as COPD and emphysema, even among non-smokers [40,41]. The mechanisms by which SHS induces lung injury involve persistent inflammatory responses, vascular dysfunction, oxidative stress, and progressive tissue remodeling [41,42]. While it is generally accepted that SHS is avoidable for most, the consequences suffered by the chronically exposed warrants helpful interventions. In this study, we characterized SHS-induced lung inflammation and dysfunction in a murine model. The data demonstrated that therapeutic targeting of bromodomain and extra-terminal (BET) proteins using VYN202, a novel oral small-molecule BET inhibitor, markedly attenuates pathological responses.

Our findings show that SHS exposure significantly elevated total protein concentration, leukocyte infiltration, and neutrophil accumulation in BALF, consistent with alveolar–capillary barrier disruption and neutrophilic inflammation [43]. Increased BALF protein and PMN percentages are hallmark features of acute and chronic smoke-induced lung injury [44]. Notably, VYN202 treatment substantially mitigated these changes, suggesting preservation of alveolar integrity and suppression of neutrophilic infiltration, two critical components of limiting early lung injury progression. A natural follow-up endeavor should include complete profiling of activated immune cells in models exposed to SHS with and without VYN202. Furthermore, such an undertaking should also include an array of pulmonary spirometry assessments in order to evaluate potential benefits of V202 in rescuing SHS-induced impairment of lung function.

We also identified marked activation of several receptor tyrosine kinases (RTKs) implicated in inflammatory amplification (JAK1, JAK3, ABL1, ACK1) following SHS exposure. RTK dysregulation promotes immune hyperactivation and sustained cytokine release, contributing to chronic airway inflammation [45]. VYN202 effectively suppressed the activation of these RTKs, suggesting that BET inhibition can modulate upstream signaling events that drive inflammation. Parallel suppression of pro-inflammatory cytokines and chemokines such as TNF-α, IL-17A, and IFN-γ by VYN202 supports this conclusion, consistent with the role of BET proteins in controlling transcriptional activation of inflammatory genes [46,47].

Secondhand smoke exposure induced a complex dysregulation of vascular homeostasis through differential effects on receptor tyrosine kinases involved in angiogenesis and vascular remodeling. Endothelial dysfunction is increasingly recognized as a central mechanism underlying cigarette smoke-induced lung injury [48]. Consistent with a pro-remodeling phenotype, SHS significantly increased phosphorylation of VEGFR3 and FAK (Figure 3), both of which have been implicated in pathologic vascular leakage, fibroblast activation, and early fibrotic processes in COPD models [49]. In contrast, SHS reduced activation of VEGFR2, EphB4, EphB6, JAK2, Tyk2, and NGFR (Figure 4), receptors that, under normal conditions, support endothelial barrier integrity and anti-inflammatory signaling. Notably, concomitant administration of VYN202 normalized nearly all of these alterations, reducing the SHS-induced upregulation of VEGFR3 and FAK while restoring activation of the downregulated vascular-protective RTKs (Figure 3 and Figure 4). These findings suggest that BET inhibition by VYN202 not only suppresses classic pro-inflammatory RTK signaling (JAK1/3, ABL1, ACK1) but also rebalances vascular RTK networks, potentially limiting early maladaptive remodeling responses to chronic smoke exposure.

Taken together, these findings highlight the complexity of SHS-induced lung pathology. This complexity includes epithelial barrier disruption, leukocyte-driven inflammation, cytokine amplification, vascular instability, and impaired immunoregulation. This research therefore demonstrates that BET inhibition via VYN202 can target multiple pathological nodes simultaneously. This broad activity profile likely stems from the central role of BET proteins in orchestrating transcriptional programs underlying both inflammatory and vascular responses [46,50].

The therapeutic potential of VYN202 is particularly noteworthy given its oral bioavailability, favorable selectivity for BD2 domains of BET proteins, and previous evidence supporting its efficacy in models of autoimmune and inflammatory diseases [46,47]. Prior BET inhibitors such as JQ1 and I-BET762 have shown promise in reducing inflammatory gene expression in lung disease models [46,51], but clinical translation has been limited by off-target effects and pharmacokinetic challenges. VYN202’s preferential BD2 binding profile may confer enhanced safety and specificity, reducing the risk of broad transcriptional repression and allowing for chronic administration if needed. These caveats should be better clarified in a subsequent study that profiles individual epithelial, endothelial, or immune populations. Such studies should focus on defining the direct transcriptional effects of BET inhibition in specific lung cell types to determine whether the observed RTK and cytokine modulation is cell-autonomous or secondary to reduced inflammation.

To contextualize VYN202’s effects, comparisons to established BET inhibitors like JQ1, which have been shown to attenuate smoke-induced pulmonary inflammation, are justified. For instance, JQ1 reduces IL-6, TNF-α, and MMP-2/9 expression in COPD models, alleviating oxidative stress and airway remodeling. Similarly, I-BET762 suppresses inflammatory gene transcription in respiratory diseases. However, these are pan-BET inhibitors targeting both BD1 and BD2 domains, potentially leading to broader transcriptional disruptions and side effects compared to VYN202’s BD2-selective profile [52]. In our SHS model, VYN202 attenuated comparable markers (e.g., TNF-α, IL-17A, JAK1) via oral administration, which is advantageous for clinical translation over the intraperitoneal or in vitro delivery often used for JQ1. While direct head-to-head studies are warranted, VYN202’s selectivity and ongoing clinical evaluation (e.g., for psoriasis) suggest it may offer a safer, more targeted option for smoke-related lung pathologies.

While our findings are promising, several limitations remain. By design, our study centered on SHS, which has variable avoidability. Specifically, the current project focused on acute SHS exposure in female mice; therefore, the long-term efficacy of VYN202 in chronic smoke models involving both sexes remains to be determined. Female mice were included based on prior evidence of more robust acute inflammatory responses in females under similar SHS conditions. A chronic course that compares SHS and primary smoking would accordingly warrant an expanded battery of tests that would more fully characterize differentiated inflammation and apoptotic networks over time. The chronic course would naturally involve clarifying emphysematous remodeling via histology, linear intercept differences, and Evans Blue or other assessments of vascular compromise. Additionally, further mechanistic studies dissecting BET-regulated transcriptional networks in specific lung cell types would provide deeper insight into how VYN202 exerts its protective effects. Whether VYN202 can suppress or reverse pre-existing, established pulmonary inflammation and early tissue remodeling remains unknown. Future therapeutic-protocol studies in which VYN202 treatment is initiated only after inflammation has been established for several weeks will be critical to determine its potential as a treatment for ongoing smoke-related lung disease. Our aim was not to model therapeutic reversal of established injury, but rather to examine whether VYN202 could prevent or blunt the initial molecular and cellular activation events that underlie SHS-induced inflammation. Additional dose–response studies and post-injury (therapeutic) paradigms are critical for understanding the full translational potential of VYN202.

## 5. Conclusions

This study identifies VYN202 as a capable inhibitor of SHS-induced pulmonary inflammation and vascular dysfunction. By simultaneously attenuating pro-inflammatory cytokine production, preserving vascular integrity, and restoring immune regulatory pathways, VYN202 offers a novel and promising therapeutic approach for mitigating smoke-induced lung disease.

## Data Availability

The raw data supporting the conclusions of this article will be made available by the authors on request.

## Supplementary Materials

The following supporting information can be downloaded at https://www.mdpi.com/article/10.3390/cimb47121062/s1.

## Abbreviations

ABL1—Abelson Tyrosine Kinase 1; ACK1—Activated Cdc42-associated Kinase 1; AGE—Advanced Glycation End-products; ARRIVE—Animal Research: Reporting of In Vivo Experiments; BALF—Bronchoalveolar Lavage Fluid; BD1—Bromodomain 1; BD2—Bromodomain 2; BET—Bromodomain and Extra-Terminal; BETi—BET Inhibitor; BRD2, 3, 4—Bromodomain-containing Protein 2, 3, 4; COPD—Chronic Obstructive Pulmonary Disease; DMSO—Dimethyl Sulfoxide; EC50—Half Maximal Effective Concentration; ECM—Extracellular Matrix; EphB4, 5—Ephrin Type-B Receptor 4, 5; FAK—Focal Adhesion Kinase; G-CSF—Granulocyte Colony-Stimulating Factor; H&E—Hematoxylin and Eosin; IFN-γ—Interferon-Gamma; IL-1β—Interleukin-1 Beta; IL-10—Interleukin-10; IL-12p70—Interleukin-12p70; IL-17A—Interleukin-17A; IL-6—Interleukin-6; JAK1—Janus Kinase 1; JAK2—Janus Kinase 2; JAK3—Janus Kinase 3; LIX—Lipopolysaccharide-Induced CXC Chemokine; NF-κB—Nuclear Factor Kappa-light-chain-enhancer of Activated B Cells; NGFR—Nerve Growth Factor Receptor; PBS—Phosphate-Buffered Saline; PEF—Peak Expiratory Flow; PMN—Polymorphonuclear Neutrophil; RA—Room Air; RAGE—Receptor for Advanced Glycation End-products; RTK—Receptor Tyrosine Kinase; SHS—Secondhand Smoke; STAT3—Signal Transducer and Activator of Transcription 3; TNF-α—Tumor Necrosis Factor-Alpha; Tyk2—Tyrosine Kinase 2; VEGFR2—Vascular Endothelial Growth Factor Receptor 2; VEGFR3—Vascular Endothelial Growth Factor Receptor 3; VYN202—Small-Molecule BET Inhibitor.
