# Supplementary material for: Potential Use of VYN202, a Novel Small Molecular Bromodomain and Extra-Terminal Inhibitor, in Mitigating Secondhand Smoke (SHS)-Induced Pulmonary Inflammation
Source: Curr Issues Mol Biol. 2025 Dec 18;47(12):1062. doi: 10.3390/cimb47121062 (PMC12731855; doi:10.3390/cimb47121062)

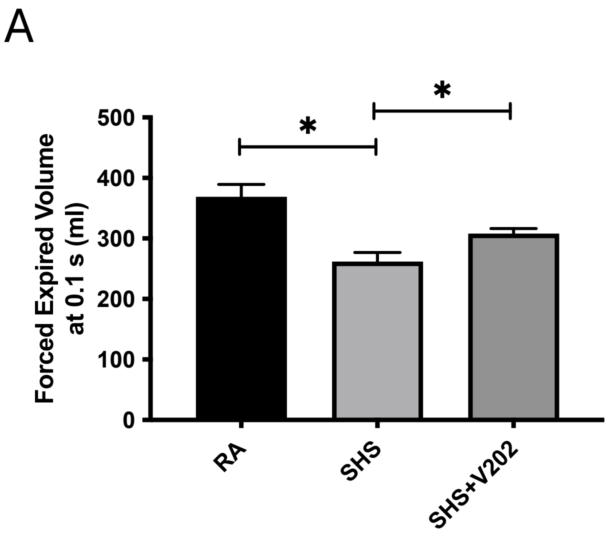

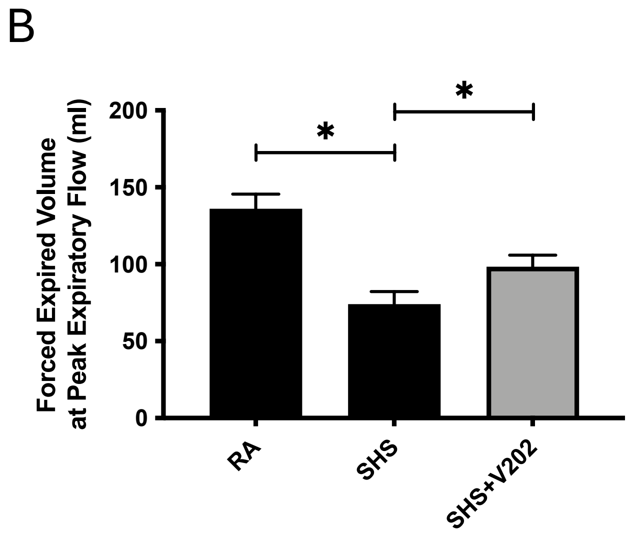

Supplementary Figure S1. Lung physiology was evaluated to determine alterations in mechanics. Mice exposed to acute SHS experienced significantly diminished FEV0.1 (Forced Expiratory Volume at 0.1 s, * $p < 0.05$) (**A**). FEV at PEF (Peak Expiratory Flow) was also significantly diminished in Mice exposed to SHS compared to RA controls ((**B**), * $p < 0.05$). Animals co-treated with VYN202 were significantly protected from SHS-induced changes in FEV0.1 (**A**), and FEV at PEF (**B**). Evaluations were obtained in mice attached to the FlexiVent Instrument (Scireq) (n = at least 6 animals per group).

Lung mechanics measurements were conducted using the flexiVent FX system (SCIREQ Inc., Montreal Qx, Canada). The instrument was equipped with a FX1 module and a Negative Pressure-Driven Forced Expiration (NPFE) extension for mice run by flexiWare 8.0 software. Mice were anesthetized using an intraperitoneal injection of ketamine-xylazine (100 and 10 mg/kg body weight) in 0.9% sterile saline. Once mice were observed to be in a surgical plane of anesthesia, the trachea was exposed to insert a 22-gauge metal cannula. The mice were then attached to the flexiVent and received an intraperitoneal injection of 0.8mg/kg body weight pancuronium bromide to prevent spontaneous breathing. The plethysmograph chamber was secured over the mice. Mice were ventilated with a tidal volume of 10 mL/kg with a frequency of 150 breaths/min and an end-expiratory pressure of 3 cmH2O. The baseline was recorded, and the following scripts were run three times: Deep Inflation, Snapshot-150, Quick Prime, Negative Pressure-Driven Forced Expiration (NPFE) as detailed by the manufacturer. Mice were euthanized after all the scripts were completed.

Representative examples of Dot Blot membranes

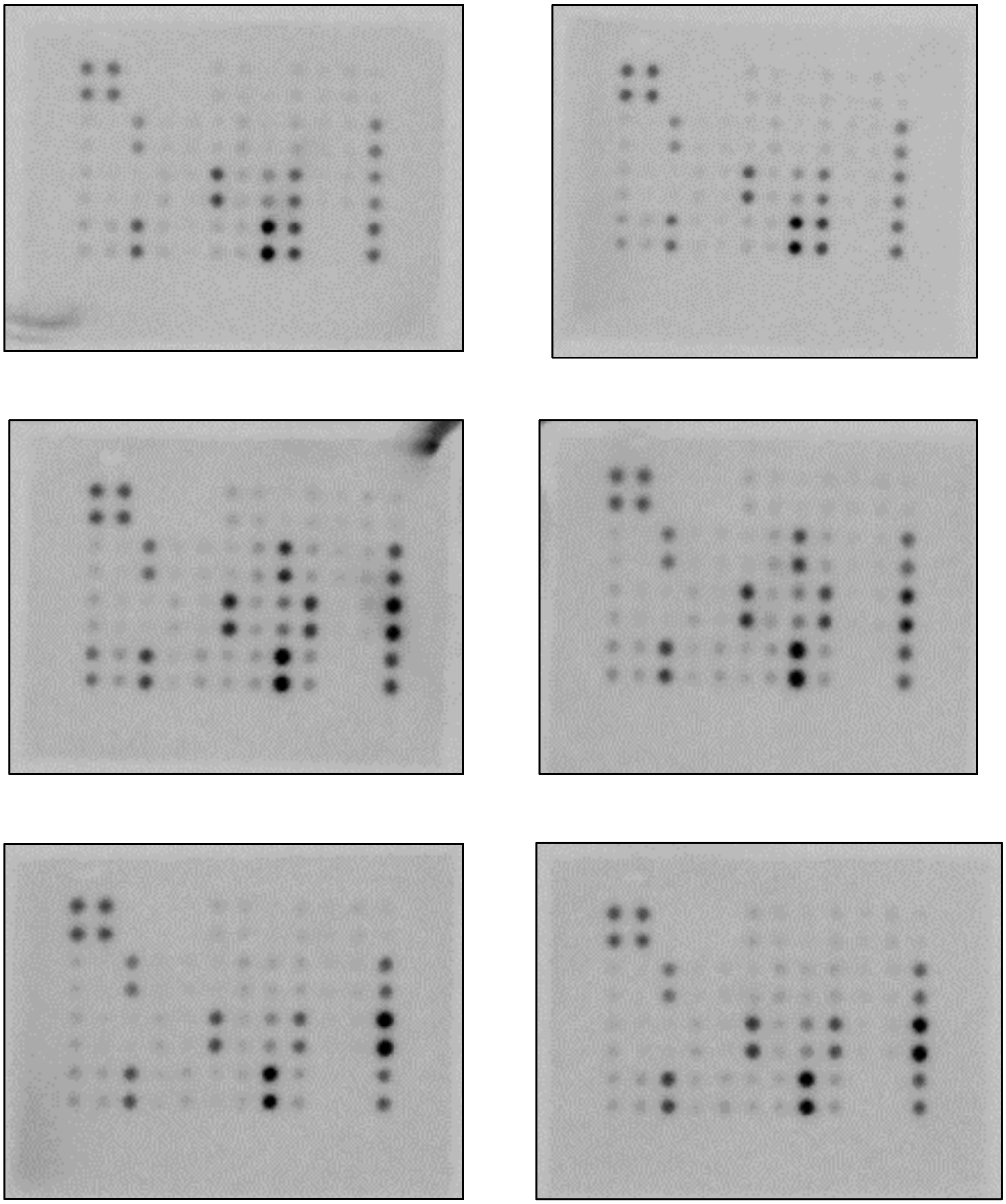

Supplement: Supplementary file 1 [file cimb-47-01062-s001.zip › cimb-3997878-supplementary.pdf]
